# Synergistic amelioration of renal oxidative stress, inflammation, and fibrosis by combination of metformin and *Clinacanthus nutans* leave extracts in a type 2 diabetic rat model

**DOI:** 10.3389/fphar.2025.1558341

**Published:** 2025-04-29

**Authors:** Pongrapee Laorodphun, Saruda Thongyim, Sureeporn Suriyaprom, Pornchita Maphet, Yingmanee Tragoolpua, Thida Kaewkod, Aussara Panya, Phatchawan Arjinajarn

**Affiliations:** ^1^ Department of Biology, Faculty of Science, Chiang Mai University, Chiang Mai, Thailand; ^2^ Office of Research Administration, Chiang Mai University, Chiang Mai, Thailand; ^3^ Graduate Master’s Degree Program in Biology, Faculty of Science, Chiang Mai University, Chiang Mai, Thailand

**Keywords:** modern medicine, traditional medicine, phenolic compounds, renal injury, type 2 diabetes

## Abstract

**Background:**

Combination treatment enhances the therapeutic potential for diabetes, particularly for patients with severe complications. Combining standard therapeutic drugs with alternative bioactive compounds provides a promising option for long-term treatment, given the high safety profile of bioactive substances. Objective in this study, we aimed to evaluate the synergistic effects of metformin and *Clinacanthus nutans* (Burm. f.) Lindau (CN) on glucose metabolism and renal dysfunction parameters in a type 2 diabetic rat model.

**Methods:**

Male Wistar rats were fed a high-fat diet for 4 weeks and then received a low dose of streptozotocin to induce type 2 diabetes. The diabetic rats were randomly divided into four groups: untreated diabetic rats (DM), diabetic rats treated with CN at doses of 100 or 200 mg/kg/day (DM100 or DM200), diabetic rats treated with a combination of CN and metformin (DMCOM), and diabetic rats treated with metformin at 100 mg/kg/day (DMMET). The treatments were administered by gavage for 4 weeks.

**Results:**

Compared to single treatments, DMCOM showed a remarkable effect in reducing several parameters, including serum creatinine and blood urea nitrogen, while enhancing creatinine clearance in diabetic rats. Additionally, DMCOM significantly decreased malondialdehyde levels. Notably, diabetic rats treated with DMCOM exhibited a significant reduction in parameters associated with renal dysfunction, as evidenced by decreased inflammation markers, along with downregulated fibrotic markers.

**Conclusion:**

Our findings provide a scientific basis for the clinical application of CN and suggest a new strategy for preventing nephrotoxicity and other kidney diseases in diabetic patients.

## Introduction

Current lifestyles are characterized by high-calorie diets disrupt energy balance, leading to the development of obesity and metabolic syndrome. Excessive fat accumulation, particularly visceral adiposity, in obesity is associated with chronic inflammation, oxidative stress, and insulin resistance. These conditions can result in severe outcomes such as pancreatic β-cell dysfunction, type 2 diabetes (T2D), and T2D-associated chronic kidney disease (CKD), which may progress to end-stage renal failure (Jin et al., 2023b).

Oxidative stress is a key factor contributing to complications in patients with T2D. Systemic oxidative stress is strongly associated with obesity, excessive consumption of high-fat diets, and obesity-related kidney diseases in both humans and animal models (Manna and Jain, 2015). In obesity, reactive oxygen species (ROS) are overproduced and play a critical role in the development of T2D and its complications. Elevated malondialdehyde (MDA) levels, a biomarker of oxidative stress, have been observed in obese patients, showing a strong correlation with body mass index (BMI), body fat percentage, and lipid profile levels (Abu Khadra et al., 2024). Diabetes-associated renal dysfunction and diabetic nephropathy are among the most common complications of T2D, both of which are closely linked to excessive oxidative stress. In response to oxidative stress, the glomerular filtration barrier a three-part system consisting of fenestrated endothelial cells, the glomerular basement membrane, and podocyte undergoes significant deterioration (Jin et al., 2023a). Additionally, the expression of nephrin, a protein biomarker indicative of podocyte function, is reduced (Sekulic and Pichler Sekulic, 2013) further contributing to renal dysfunction. Moreover, hyperglycemia and hyperlipidemia in T2D have been shown to enhance the production of superoxide anions, hydroxyl radicals and hydrogen peroxide from renal cortical mitochondria (Jha et al., 2016). These ROS induce cell injury and death, which reflects both its impaired structure and function.

Another key factor contributing to renal dysfunction and diabetic nephropathy is endoplasmic reticulum stress (ER stress) (Fan et al., 2017) demonstrated that the ER stress induced by T2D triggering the unfolded protein response (UPR) activation. This activation initiated the synthesis of the ER chaperone GRP78, also known as BiP, a 78-kDa glucose-regulated protein that regulates the endoplasmic reticulum (ER) stress response (Li et al., 2008). Furthermore, ER stress induces the activation of calpain, which subsequently activates caspase-12 (Morishima et al., 2002; Xie et al., 2020), a process driven by increased cytosolic calcium levels. In addition, sustained or severe ER stress enhances the expression of the transcription factor C/EBP homologous protein (CHOP) (Hu et al., 2018), which subsequently promotes the activation of NF**κ**B and caspase. Excessive ER-stress response might cause significant renal injury, ultimately leading to renal dysfunction (Cheng et al., 2024).

Metformin has been the first-line treatment for T2D for decades; however, the progressive loss of β-cells makes it challenging for diabetes patients to achieve glycemic control with monotherapy alone, highlighting the need for combination therapy to manage disease progression and reduce the risk of serious complications (Baker et al., 2021). Furthermore, as with other oral antidiabetic medications, high rates of secondary failure have been reported following initially successful metformin therapy (Halimi et al., 2008). Therefore, a combination approach with other substances that provide potential direct effects on antioxidant properties could enhance anti-diabetic activity and help prevent renal dysfunction and diabetic nephropathy. *Clinacanthus nutans* (Burm. f.) Lindau (CN) has been reported for its high safety profile based on traditional use and its broad activities, including antioxidant, anti-inflammatory, anti-bacterial, anti-viral, and anti-diabetic effects (Umar Imam et al., 2019). Moreover, the potential antioxidant activity of CN has been reported to inhibit α-glucosidase and DPP-IV enzymes, contributing to improved glucose levels. Given that CN extract is known for its antioxidant and anti-diabetic properties, the present study aimed to investigate the synergistic effects of metformin and CN extract on glucose metabolism, as well as their roles in reducing inflammation, improving antioxidant status, and alleviating ER stress in diabetic rats.

## Materials and methods

### Preparation and extraction of *Clinacanthus nutans* (Burm.f.) lindau

Fresh leaves of *Clinacanthus nutans* (Burm.f.) Lindau were sourced from Lampang Province, northern Thailand. Botanical identification was conducted by Dr. Wittaya Pongamornkul, and a voucher specimen (WP9182) was deposited at the Queen Sirikit Botanic Garden Herbarium (QSBG). The leaves were dried in an oven at 60°C (Binder, Tuttlingen, Germany), ground into a fine powder using a blender, and extracted with 95% ethanol at a 1:10 (w/v) ratio (Sai-Ut et al., 2023). Specifically, 1 kg of plant powder was extracted with 10 L of 95% ethanol.

The extraction process involved shaking the mixture on an orbital shaker (IKA, Staufen, Germany) at 150 rpm for 72 h at room temperature. After the first extraction, the solvent was separated, and the plant material was subjected to a second round of extraction under identical conditions with an additional 10 L of 95% ethanol. The combined extracts were passed through Whatman No. 1 filter paper (Cytiva, Marlborough, MA, United States) and concentrated under reduced pressure with a rotary evaporator (Heidolph, Schwabach, Germany) at 45°C. The concentrated extract was then freeze-dried using lyophilization equipment (LABCONCO, Kansas City, MO, United States) to obtain the final product.

### Cytotoxicity

To determine the cytotoxicity of *CN* crude extracts, the cell viability of RAW 264.7 cell lines was examined after treatment with the extract at the concentrations of 0.8–2,500 μg/mL (5-fold dilution). Briefly, RAW 264.7 cells (100,000 cells/well) were seeded a day before the experiment in the 96-well plates. The RAW 264.7 cell viability were determined after CN treatment for 24 and 48 h by using the PrestoBlue™ cell viability reagent (Invitrogen, Massachusetts, United States). The absorbance at OD570 and OD595 was monitored by a microplate reader and used to calculated the percentage of cell viability (% cell viability) relative to that of the control (non-treated cells) using the formula below.

$$\text{The }\%\text{ cell viability}=\frac{\left(\text{OD}570-\text{OD}595\right)\;\text{treated}\;\text{cells}}{\left(\text{OD}570-\text{OD}595\right)\;\text{non}‐\text{treated}\;\text{cells}}\;x\;100$$

## Animals

The male Wistar rats, which had an average weight of 180–200 g, were procured from Nomura Siam International, a supplier located in Bangkok, Thailand. A 7-day period is necessary for the creatures to adjust to their unusual environment. The rats used as experimental subjects were confined in a controlled environment that adhered to specific conditions. The conditions specified were a 12-h light/dark cycle, a humidity level of 55%, and a constant temperature of 25 °C. The rodents were provided with unlimited access to distilled water and a standard pellet diet during the acclimatization phase. The laboratory animal center at Chiang Mai University in Chiang Mai, Thailand, has granted sanction (Permit Number: 2566/RT-0009) for the animal facilities and procedures utilized during this investigation.

### Experimental design

**FIGURE 1:**
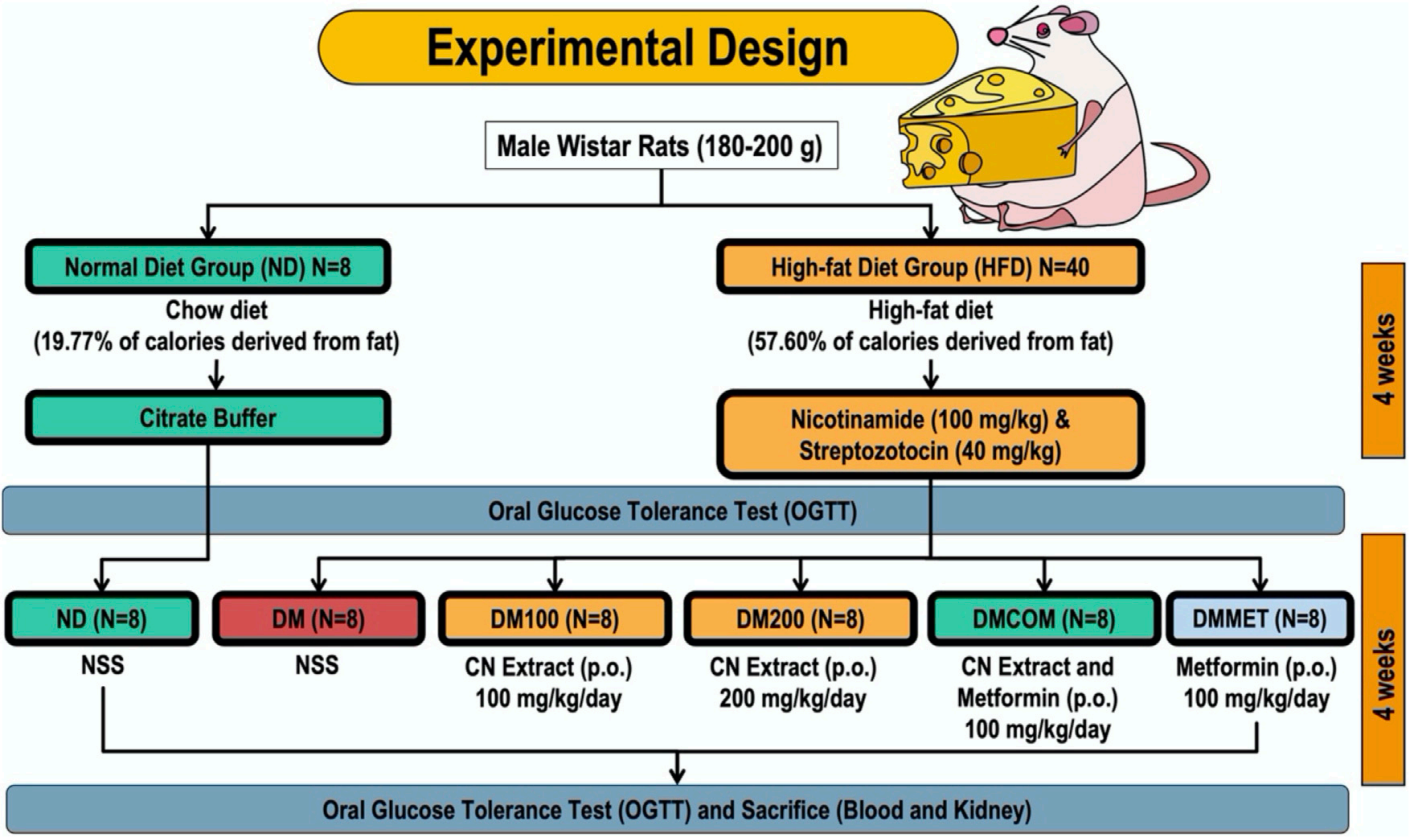
Rat experimental timeline.

The rodents were randomly divided into two dietary groups: a normal diet (ND) group and a high-fat diet (HFD) group. Eight rats in the ND group were fed a standard chow diet (C.P. Mice Feed Food No. 082) containing 19.77% of calories from fat. Meanwhile, the HFD group (n = 40) was provided a high-fat diet, where 57.60% of the caloric intake was derived from fat. The rats consumed the high-fat diet for 4 weeks before inducing diabetic impairment. This was achieved by administering a high-fat meal followed by an intraperitoneal injection of nicotinamide (100 mg/kg) and a low dose of streptozotocin (STZ) (40 mg/kg) to induce type 2 diabetes (T2D). In contrast, the control group received citrate buffer. The ND-fed rats were fed with vehicle (NSS). The oral glucose tolerance test (OGTT) confirmed the model’s validity. Similarly, the HFD-fed rodents were split into five subgroups, each comprising eight rats. The following groups were treated: diabetic rats receiving a vehicle (referred to as DM); diabetic rats treated with CN extract (referred to as DM100, administered at a dosage of 100 mg/kg/day); diabetic rats treated with CN extract (referred to as DM200, administered at a dosage of 200 mg/kg/day); diabetic rats treated with a combination of CN extract and metformin (referred to as DMCOM, administered at a dosage of 100 mg/kg/day); and diabetic rats treated with metformin alone (referred to as DMMET, administered at a dosage of 100 mg/kg/day). The gavage technique used to administer CN extract and metformin orally for a period of 4 weeks, following their immediate dissolution in normal saline (NSS) (Figure 1). The energy intake was calculated using the following equations:

$$\text{Energy intake of ND }\left(\text{Kcal}/\text{day}\right)=\text{food intake }\left(g/\text{day}\right)\times \text{constant of standard chow }\left(4.2\text{ kcal}/g\right)$$

$$\text{Energy intake of HF }\left(\text{Kcal}/\text{day}\right)=\text{food intake }\left(g/\text{day}\right)\times \text{constant of high}‐\text{fat diet }\left(5.35\;\text{kcal}/g\right)$$

### Oral glucose tolerance test (OGTT)

The OGTT was administered during the fourth and eighth weeks. After an overnight period of fasting, the rodents were administered a glucose solution (2 g/kg) via gavage. The subjects were anesthetized during the pre-glucose loading phase (0 min) and the post-glucose injection periods of 30, 60, and 120 min, during which blood samples were collected from the distal end of the tail at various time intervals. Blood glucose levels were assessed using colorimetric test devices. The organization in question is Erba Diagnostics Mannheim GmbH, which is situated in Mannheim, Germany. Subsequently, the trapezoidal formula was utilized to assess impaired glucose tolerance. This involved calculating the area under the curve for glucose levels during the OGTT, which is commonly referred to as TAUCg.

### The homeostasis model assessment (HOMA) index

The fasting plasma glucose and plasma insulin levels were utilized to calculate the homeostasis model assessment (HOMA) index in the following manner:

$$\text{HOMA}‐\text{IR}=\left[\text{Fasting insulin level }\left(\text{ng}/\text{mL}\right)\;x\text{ Fasting glucose level }\left(\text{mg}/\text{dL}\right)\right]/22.5$$

Insulin resistance or low insulin sensitivity is indicated by a high HOMA-IR value, while high insulin sensitivity is indicated by a low HOMA-IR reading.

### Blood and renal tissue sampling

Each OGTT trial was preceded by the collection of 24-h urine samples using individual metabolic chambers. Following this, blood samples were drawn from the abdominal aorta after the animals were euthanized with an overdose of 2% isoflurane anesthesia. Serum and plasma samples were separated prior to use and stored at −20°C. The kidneys were then promptly removed, cleared of surrounding tissues, and weighed. For histological analysis, the kidneys were sectioned into two parts. One portion was fixed in a freshly prepared 4% paraformaldehyde solution (pH 7.4). The final step involved isolating the renal cortex, which was preserved at −80°C. The preserved cortical tissue was subsequently utilized to measure glutathione (GSH) content and malondialdehyde (MDA) levels.

### Biochemical analysis

#### Blood parameter assessment

The concentrations of triglyceride, cholesterol, and fasting plasma glucose were quantified using a colorimetric test kit (Erba Diagnostics Mannheim GmbH, Mannheim, Germany). An automated chemical analyzer (Sysmex BX-3010, Gobe, Japan) was used to quantify plasma HDL. The standard formula was utilized to determine the LDL.

$$\text{LDL}‐c=\text{total cholesterol}–\text{HDL }–\;\left(\text{triglyceride}/5\right)$$

### Renal function assessment

An automated analyzer was used to ascertain serum and urine creatinine, blood urea nitrogen (BUN), and urine protein levels (Sysmex BX-3010), Gobe, Japan.

### Determination of renal oxidative stress

The quantity of MDA in the renal cortical tissue was determined using a thiobarbituric acid reactive substances (TBARS) test kit (Cayman Chemical Company, Ann Arbor, Michigan, United States). The level of GSH/GSSG in the renal cortex was measured using a commercial assay reagent (EGTT-100, Bioassay Systems, Hayward, California, United States) in accordance with the manufacturer’s instructions.

### Western blotting

Renal cortical tissues underwent standard Western blotting procedures. Kidney cortex samples were sliced and homogenized with mammalian lysis solution (Sigma-Aldrich, St. Louis, MO, United States) to prepare whole-cell kidney lysates, supplemented with a protease inhibitor (Roche, Indianapolis, IN, United States). The lysates were centrifuged at 10,000 g for 15 min at 4°C. Equal volumes of protein extracts were loaded onto 10% or 12% sodium dodecyl sulfate-polyacrylamide gels (SDS-PAGE), electrophoresed, and transferred onto 0.2 μm polyvinylidene fluoride (PVDF) membranes (Bio-Rad, PA, United States). Membranes were then blocked for 1 h at room temperature using 5% BSA in PBS or TBS.

Primary antibodies were applied to the membranes and incubated overnight at 4 °C. These included anti-KIM-1, GRP78, iNOS, COX2 and nephrin antibodies from Abcam (Cambridge, United Kingdom), caspase-12 and TNFα antibodies from Millipore (Millipore, MA, United States), and anti-calpain-2, CHOP, p-NF-κB and TGF-β antibodies from Cell Signaling Technology (MA, United States). Anti-IL-6 were purchased from Santa Cruz Biotechnology (Santa Cruz, CA, United States). Following this, membranes were washed and incubated with HRP-conjugated anti-mouse or anti-rabbit secondary antibodies (Amersham, IL, United States) for 1 h at room temperature. Proteins were detected using enhanced chemiluminescence (ECL) and visualized with the ChemiDoc imaging system (Image Quant LAS500, GE Healthcare Limited, Buckinghamshire, United Kingdom). Band intensities were quantified using ImageJ software (National Institutes of Health, Bethesda, MD, United States), with GAPDH serving as loading controls.

### Histological examination

After 24 h of immersion in a 4% solution of fresh paraformaldehyde (pH 7.4), the kidneys were prepared for preservation. As a result, the preserved kidneys were embedded in paraffin for histological analysis. Slices of 5 μm thickness were achieved by sectioning the kidneys. Following this, the slices were counterstained with hematoxylin and eosin (H&E) and analyzed by an observer who was unaware of the treatment groups using an Olympus light microscope (×40 magnification) (Olympus America Inc., New York, United States). On the basis of the subsequent semi-quantitative assessment system. For renal injury scoring, the frequency of staining was determined using the following scale: 0 = no or hardly any cells positive, 1 = small fraction of cells positive, 2 = approximately half of the cells positive, 3 = more than half of the cells positive, 4 = all or the majority of cells positive (n = 5 fields/group), cells positive included macrophage infiltration, renal tubular dilation, Bowman’s capsule dilation, periglomerular and interstitial fibrosis (Laorodphun et al., 2021), the kidney injury score was assessed. This investigation employed periodic acid-Schiff (PAS) and Masson’s trichrome staining techniques. To evaluate the presence of glycogen accumulation, ascertain the glomerulosclerotic index, and investigate the deposition of collagenous connective tissue fibers, these stains were applied to kidney slides.

### Statistical analysis

The statistical analysis was performed using GraphPad Prism version 8 (GraphPad Software, La Jolla, CA, United States). The mean ± standard error of the mean (SEM) format was employed to illustrate the data. The statistical methods applied in this study encompassed the application of an independent sample t-test was used to compare the differences between two groups and Fisher’s Least Significant Difference (LSD) *post hoc* tests and analysis of variance (ANOVA) were implemented in this investigation. The statistical significance of the observed disparities was assessed using these methods. A significance threshold of *p* < 0.05 was employed to ascertain statistical significance.

## Results

### Low cytotoxicity of *Clinacanthus nutans* (CN) extracts

**FIGURE 2:**
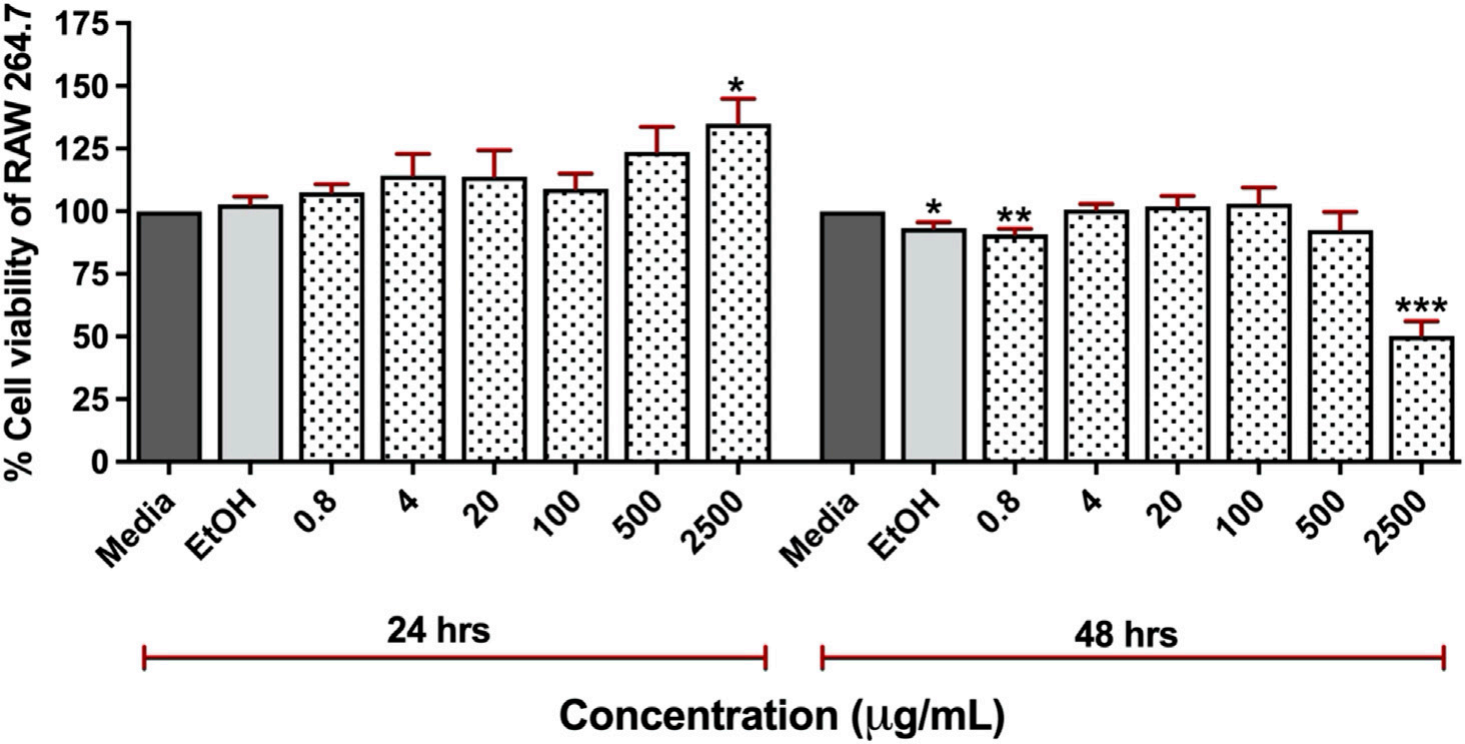
Cytotoxicity of 95% ethanolic extracts of *Clinacanthus nutans* in RAW 264.7 cells at a concentration of 0.8 μg/mL to 2,500 μg/mL for 24 and 48 h using Presto BLUE™ cell viability reagent (ns) Indicates *p* < 0.1234, *p < 0.0332, **p < 0.0021, ***p < 0.0002, and ****p < 0.0001.

We investigated the cytotoxicity of CN extract to RAW 264.7 cells. The cells were treated with the various concentrations of CN crude extracts (0.8–2,500 μg/mL) for 24 and 48 h (Figure 2). The result showed that the CN extract was highly safe, as evidenced by no significant toxicity to the cells at the concentrations up to 500 μg/mL. At the concentrations of 0.8, 4, 20, 100, and 500 μg/mL, the cell viability at 24 h was retained to 107.5%, 114.25%, 113.75%, 109%, and 123.75% while after 48 h, it was 90.75%, 100.5%, 102%, 103%, and 92.5%, respectively. The highest tested concentration (2,500 μg/mL) exhibited significant toxicity after 48 h of treatment (50.25%) but no significant effect at 24 h of incubation. Furthermore, we have determined the cytotoxicity of CN extract in human cell lines, HEK293T cells. The result showed that HEK293T cells were more sensitive to CN extract the CC50 of 327.3 μg/mL and 644.4 μg/mL at 24 h and 48 h (Supplementary Figure S1). Based on these findings, we used concentrations lower than 300 μg/mL for subsequent experiments in animal model.

### Effect of extract and metformin combination on metabolic status

**FIGURE 3:**
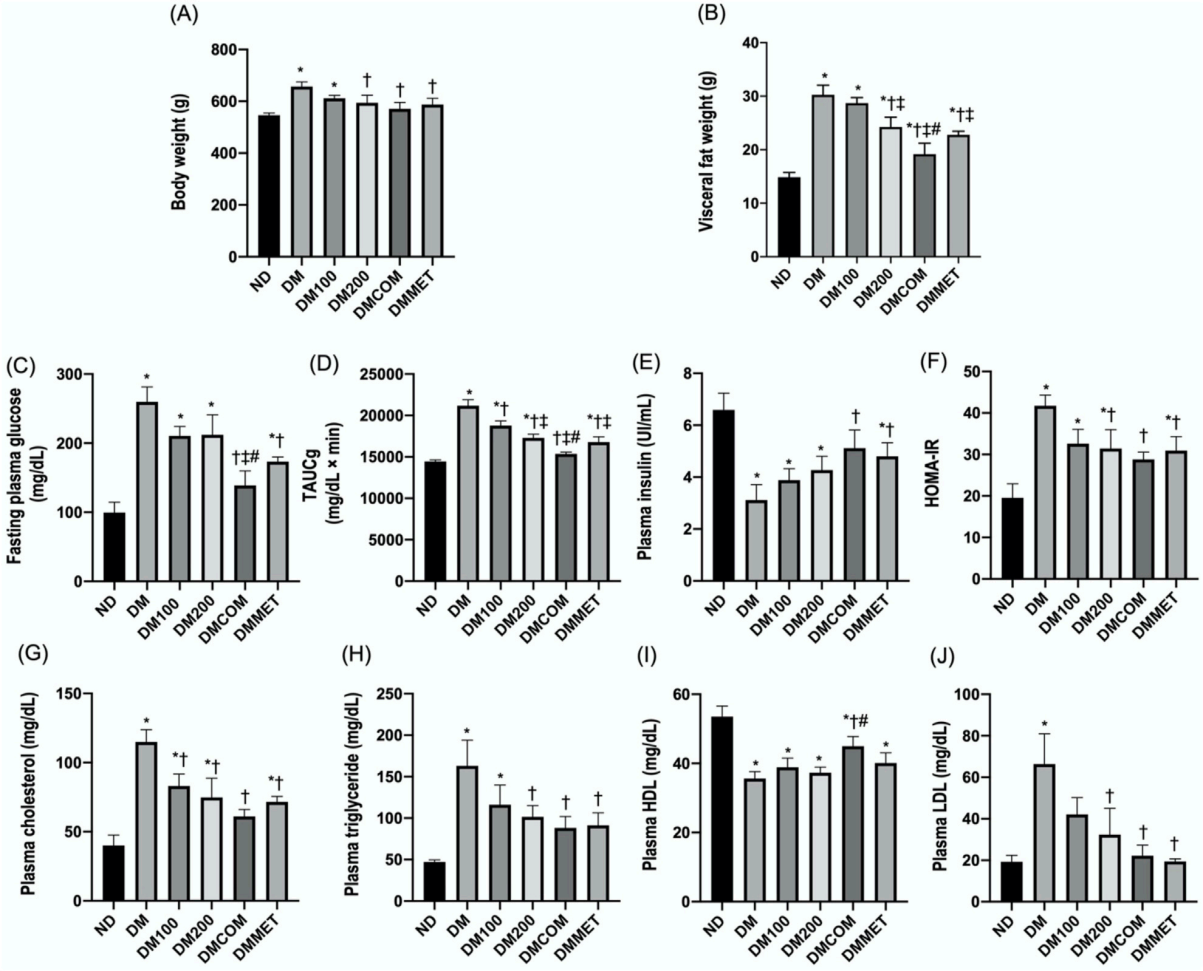
Effect of extract and metformin combination on metabolic status. Body weight **(A)**, visceral fat weight **(B)**, fasting plasma glucose **(C)**, TAUCg **(D)**, plasma insulin **(E)**, HOMA-IR **(F)**, plasma cholesterol **(G)**, plasma triglyceride **(H)**, plasma HDL **(I)**, and plasma LDL **(J)**. ND: normal rats treated with vehicle; DM: diabetic rats treated with vehicle; DM100: diabetic rats treated with CN extract 100 mg/kg/day; DM200: diabetic rats treated with CN extract 200 mg/kg/day; DMCOM: diabetic rats treated with CN and metformin 100 mg/kg/day; DMMET: diabetic rats treated with metformin 100 mg/kg/day. Values with different superscript letters *p < 0.05 versus ND; ^†^ *p* < 0.05 versus DM; ^‡^ *p* < 0.05 versus DM100; ^#^ *p* < 0.05 versus DM200 (LSD test, *p* < 0.05).

To study the effect of extract and metformin, we induced diabetes in male Wistar rats using high fat diet. After 8 weeks of induction, the diabetic (DM) group exhibited significantly elevated levels of body weight, visceral fat weight, fasting plasma glucose, total area under the curve for glucose (TAUCg), HOMA-IR, plasma cholesterol, plasma triglyceride, and low-density lipoprotein (LDL) compared to the control group (ND) (Figures 3A–D, F-H, and 3J) (*p* < 0.05). In contrast, plasma insulin and HDL levels were significantly lower in DM (*p* < 0.05) (Figures 3E, I). Interestingly, the DM rats that received CN extract at doses of 100 mg/kg/day combined with metformin at doses of 100 mg/kg/day (DMCOM groups) showed significant improvements in metabolic parameters as evidenced by the changes of plasma insulin (Figure 3E), and HDL levels (Figure 3I). Additionally, in DMCOM groups, body weight, visceral fat weight, fasting plasma glucose, TAUCg, HOMA-IR, plasma cholesterol, plasma triglyceride, and plasma LDL exhibited a declining trend (Figures 3A–D, F-H, and 3J). For kidney function parameters, the DM group showed elevated levels of serum creatinine and blood urea nitrogen (BUN) compared to rats fed a normal diet (ND) (*p* < 0.05) (Table 1). However, after CN treatment, serum BUN and creatinine levels were considerably lower in the DMCOM group compared to the DM group. Moreover, the DMCOM group demonstrated a significant reduction in urinary protein levels compared to the DM group (*p* < 0.05) (Table 1).

**TABLE 1:** Renal functions in male wistar rats at 8 weeks.

Parameters	ND	DM	DM100	DM200	DMCOM	DMMET
BUN (mg/dL)	11.44 ± 0.62	17.48 ± 0.37^*^	16.94 ± 0.59^*^	15.71 ± 0.80^*^	14.12 ± 0.84^*^‡	14.92 ± 0.86^*^†
Cr (mg/dL)	0.61 ± 0.02	0.95 ± 0.07^*^	0.68 ± 0.02†	0.66 ± 0.01†	0.63 ± 0.01†	0.66 ± 0.03†
U.Cr (mg/dL)	116.89 ± 1.54	85.05 ± 8.06^*^	95.76 ± 10.92^*^	95.24 ± 5.58^*^	110.97 ± 2.75†	104.26 ± 1.31†
U.Protein (mg/dL)	111.40 ± 9.30	171.36 ± 4.98^*^	161.56 ± 20.97^*^	144.88 ± 12.67	131.88 ± 10.12†	150.84 ± 15.14^*^

Data are presented as mean ± standard error of mean (SEM, n = 5 rats/group) ND: normal rats treated with vehicle; DM: diabetic rats treated with vehicle, DM100: diabetic rats treated with CN, extract 100 mg/kg/day; DM200: diabetic rats treated with CN, extract 200 mg/kg/day; DMCOM: diabetic rats treated with CN, and metformin 100 mg/kg/day; DMMET: diabetic rats treated with metformin 100 mg/kg/day. Values with different superscript letters *p < 0.05 versus ND; †*p* < 0.05 versus DM; ‡ *p* < 0.05 versus DM100 (LSD, test, *p* < 0.05).

### The combination effects of CN extract and metformin on renal oxidative stress

**FIGURE 4:**
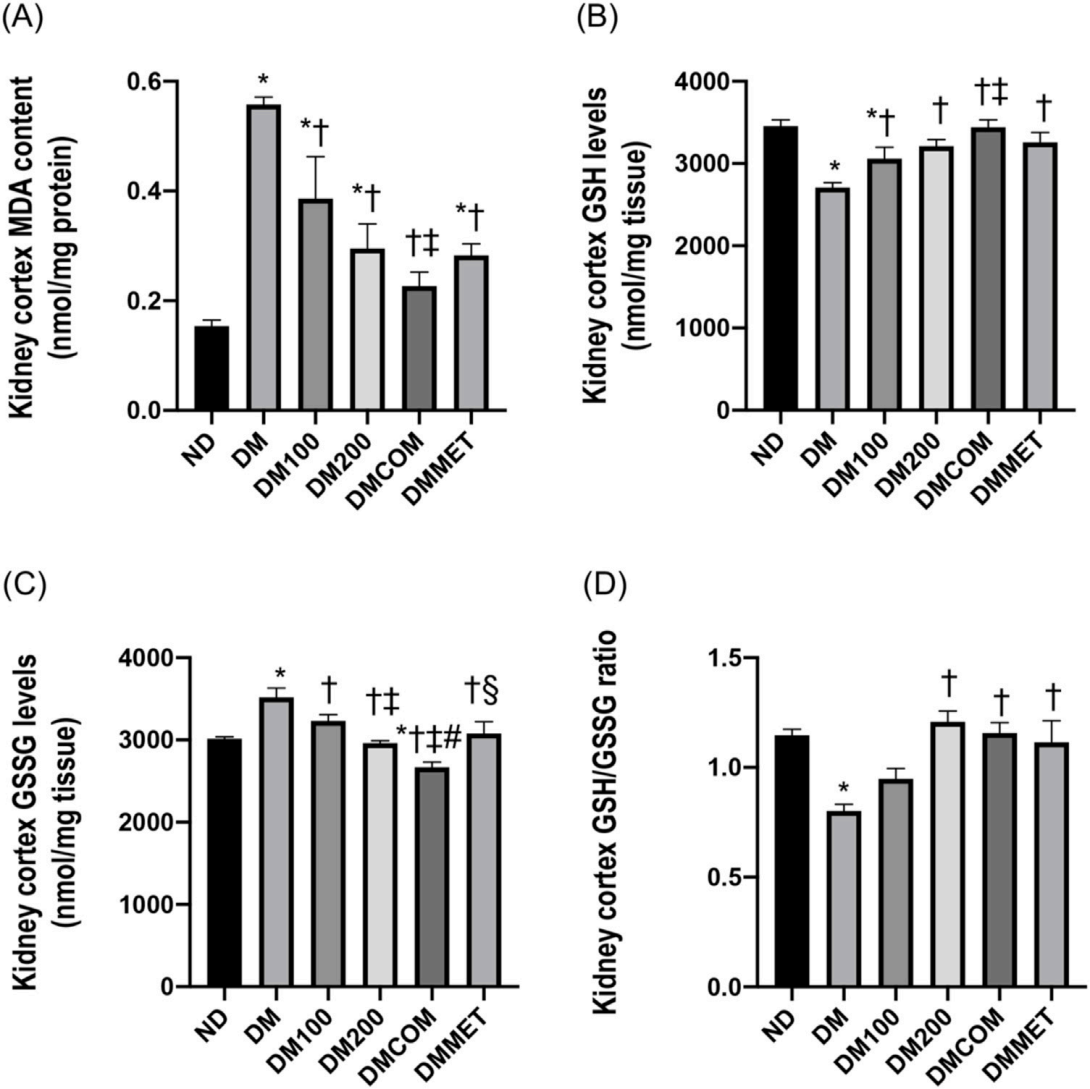
The combination effects of CN extract and metformin on renal oxidative stress. Renal cortical MDA content **(A)**, renal cortical GSH levels **(B)**, renal cortical GSSG levels **(C)**, and renal cortical GSH/GSSG ratio **(D)**. ND: normal rats treated with vehicle; DM: diabetic rats treated with vehicle; DM100: diabetic rats treated with CN extract 100 mg/kg/day; DM200: diabetic rats treated with CN extract 200 mg/kg/day; DMCOM: diabetic rats treated with CN and metformin 100 mg/kg/day; DMMET: diabetic rats treated with metformin 100 mg/kg/day. Values with different superscript letters *p < 0.05 versus ND; ^†^ *p* < 0.05 versus DM; ^‡^ *p* < 0.05 versus DM100; ^#^ *p* < 0.05 versus DM200; ^§^ *p* < 0.05 versus DMCOM (LSD test, *p* < 0.05).

Malondialdehyde (MDA), a byproduct of lipid peroxidation, is a biomarker for oxidative stress in DM rats. The DM group demonstrated a statistically significant increase in MDA levels compared to the ND group (*p* < 0.05) (Figure 4A). Similarly, the antioxidant GSH level in the renal cortex was remarkably lower in the DM group (*p* < 0.05) (Figure 4B), while the oxidized glutathione (GSSG) level was significant higher in the DM group than in the ND group (*p* < 0.05) (Figure 4C). The combination treatment with CN extract and metformin in DMCOM group improved the oxidative stress by significantly increasing GSH level and decreasing GSSG levels (*p* < 0.05) (Figures 4B, C). This suggests that the combination of CN extract and metformin has the potential to reduce oxidative stress and scavenge ROS, as supported by the significantly higher ratio of GSH/GSSG in the DMCOM group compared to the DM group (*p* < 0.05) (Figure 4D).

### The combination effects of CN extract and metformin on renal endoplasmic reticulum stress

**FIGURE 5:**
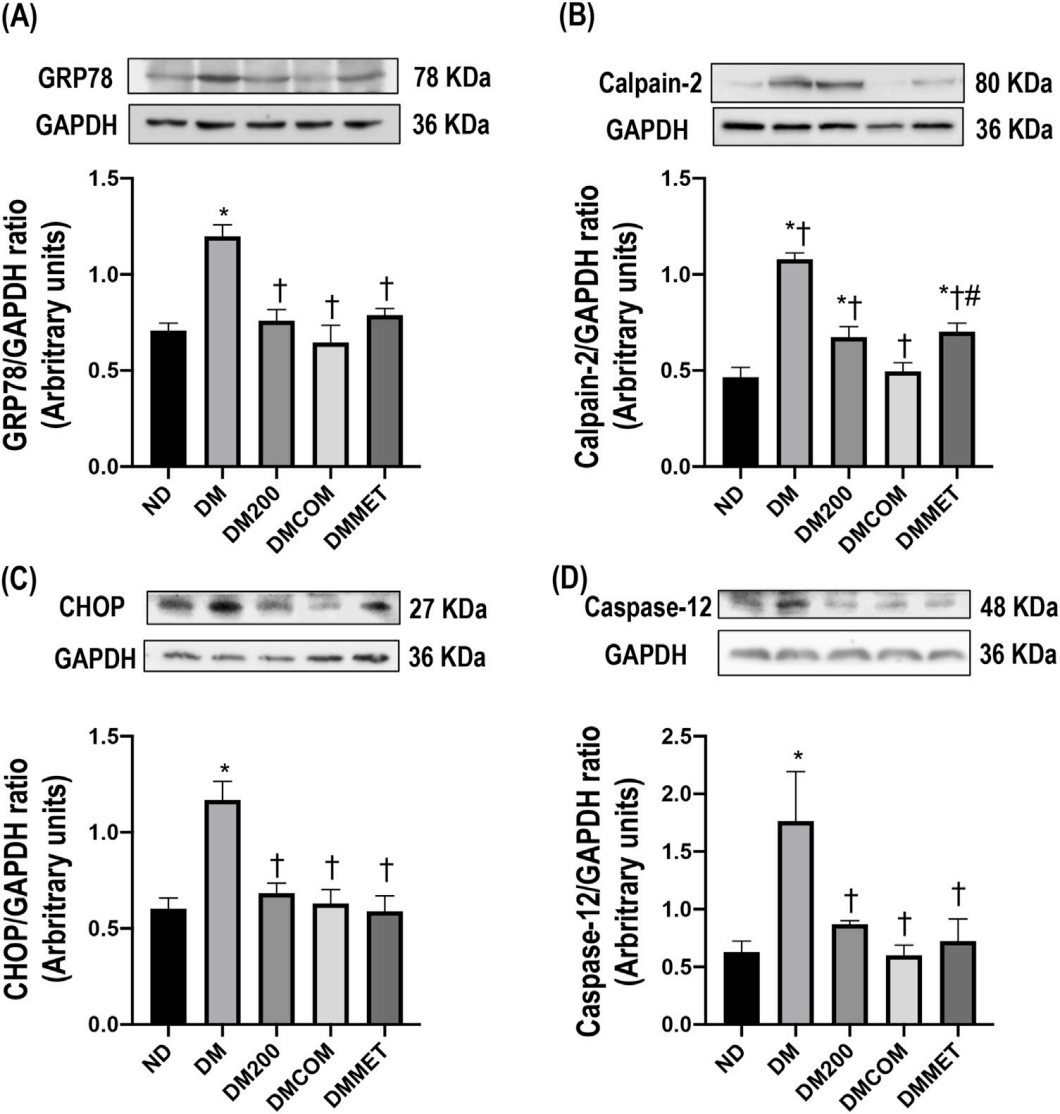
The combination effects of CN extract and metformin on renal endoplasmic reticulum stress. Renal cortical GRP78 expression **(A)**, renal cortical calpain-2 **(B)**, renal cortical CHOP **(C)**, and renal cortical caspase-12 **(D)**. ND: normal rats treated with vehicle; DM: diabetic rats treated with vehicle; DM200: diabetic rats treated with CN extract 200 mg/kg/day; DMCOM: diabetic rats treated with CN and metformin 100 mg/kg/day; DMMET: diabetic rats treated with metformin 100 mg/kg/day. Values with different superscript letters *p < 0.05 versus ND; ^†^ *p* < 0.05 versus DM; ^‡^ *p* < 0.05 versus DM200; ^#^ *p* < 0.05 versus DMCOM (LSD test, *p* < 0.05).

The endoplasmic reticulum (ER) is a subcellular target of toxic compounds that contribute to the production of reactive oxygen species (ROS) and subsequent oxidative stress, both of which are critical factors in ER stress. Our investigation focused on the alterations in proteins associated with ER stress following DM treatment. Four ER stress marker proteins including GRP78, CHOP, calpain-2, and caspase-12 were determined and compared among the treated groups. Accompanying the oxidative stress, GRP78, CHOP, calpain-2, and caspase-12 were significantly elevated in renal cortical protein expressions in the DM group compared to the ND group suggesting ER stress induced by the high-fat diet (*p* < 0.05) (Figures 5A–D). Interestingly, combination treatment with CN extract and metformin remarkably lowered all ER stress markers, suggesting that the activation of ER stress in the DM groups was effectively inhibited by combination treatment (Figures 5A–D).

### The combination effects of CN extract and metformin on renal inflammation, morphology, and fibrosis

**FIGURE 6:**
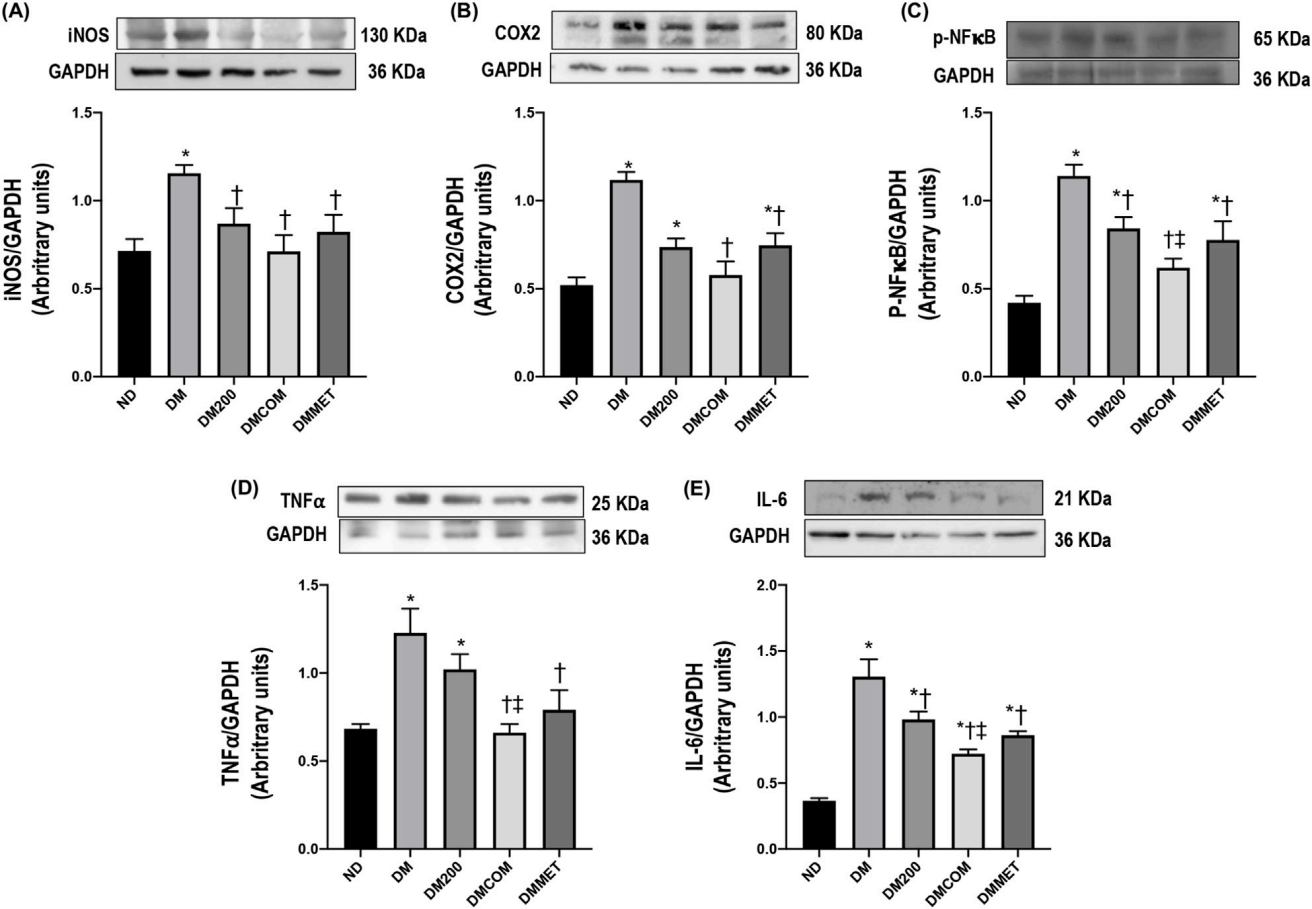
The combination effects of CN extract and metformin on renal inflammation. Renal cortical iNOS expression **(A)**, renal cortical COX2 **(B)**, renal cortical p- NF-κB **(C)**, renal cortical TNFα **(D)**, and renal cortical IL-6 **(E)**. ND: normal rats treated with vehicle; DM: diabetic rats treated with vehicle; DM200: diabetic rats treated with CN extract 200 mg/kg/day; DMCOM: diabetic rats treated with CN and metformin 100 mg/kg/day; DMMET: diabetic rats treated with metformin 100 mg/kg/day. Values with different superscript letters *p < 0.05 versus ND; ^†^ *p* < 0.05 versus DM; ^‡^ *p* < 0.05 versus DM200 (LSD test, *p* < 0.05).

We further investigated the inflammation status, as the correlation between obesity and inflammation is well documented. The expression levels of inflammatory cytokines, including iNOS, COX-2, p-NF-κB, TNFα, and IL-6, were assessed in kidney. The results indicated that DM rats exhibited significant higher expression of iNOS, COX-2, p-NF-κB, TNFα, and IL-6 compared to ND rats (*p* < 0.05) (Figures 6A–E). Together with other previous analyzed parameters, the finding demonstrated that the treatment effectively mitigated the renal inflammation, particularly the combination treatment of CN extract and metformin. The significant reduction in iNOS, COX-2, p-NF-κB, TNFα, and IL-6 expression following the treatment strongly suggests a protective effect of combination therapy against renal inflammation.

**FIGURE 7:**
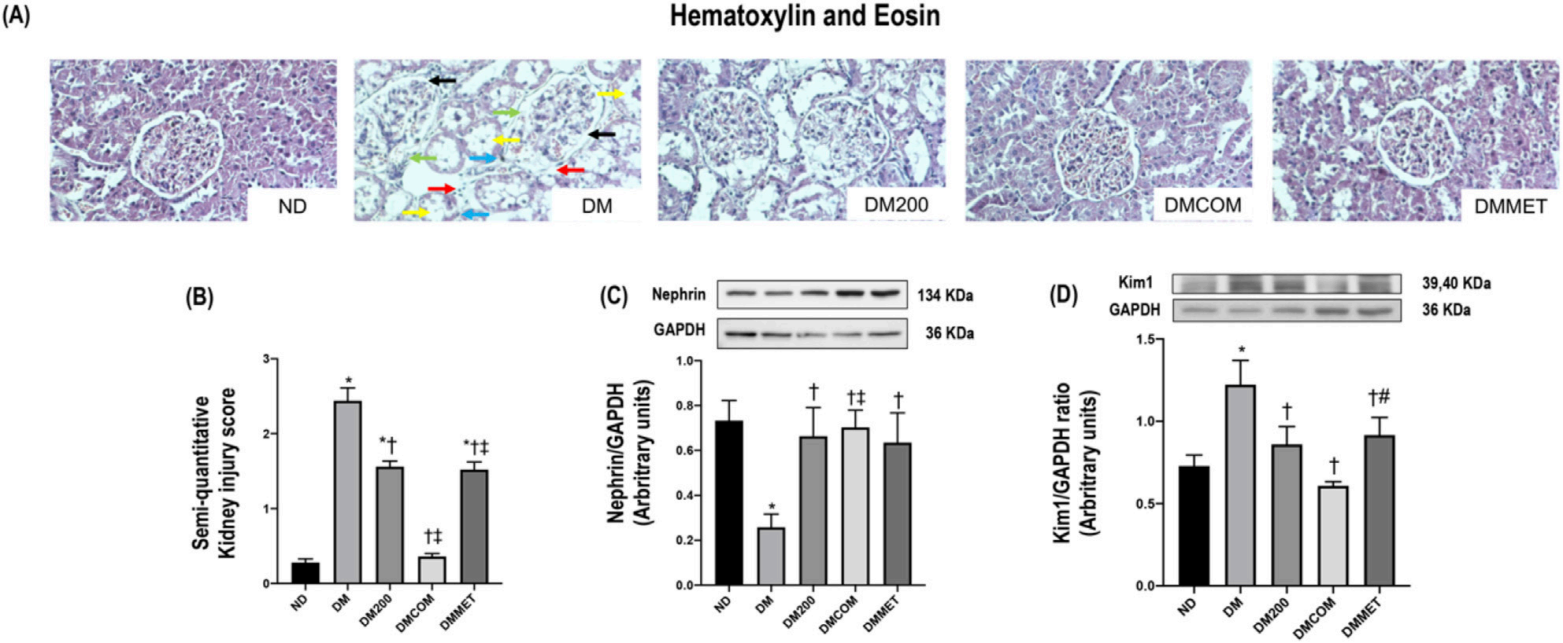
Photomicrograph histological section of kidney using hematoxylin and eosin (H&E) stain (x40) in obese rats **(A)**: Dilation of Bowman’s capsule (black arrow), interstitial fibrosis (light blue arrow), macrophage infiltration (red arrow), periglomerular fibrosis (green arrow) and tubular dilation (yellow arrow), Semi-quantitative kidney injury score **(B)**, renal cortical expression of nephrin **(C)**, renal cortical expression of KIM-1 **(D)**. ND: normal rats treated with vehicle; DM: diabetic rats treated with vehicle; DM200: diabetic rats treated with CN extract 200 mg/kg/day; DMCOM: diabetic rats treated with CN and metformin 100 mg/kg/day; DMMET: diabetic rats treated with metformin 100 mg/kg/day. Values with different superscript letters *p < 0.05 versus ND; ^†^ *p* < 0.05 versus DM; ^‡^ *p* < 0.05 versus DM200 (LSD test, *p* < 0.05).

**FIGURE 8:**
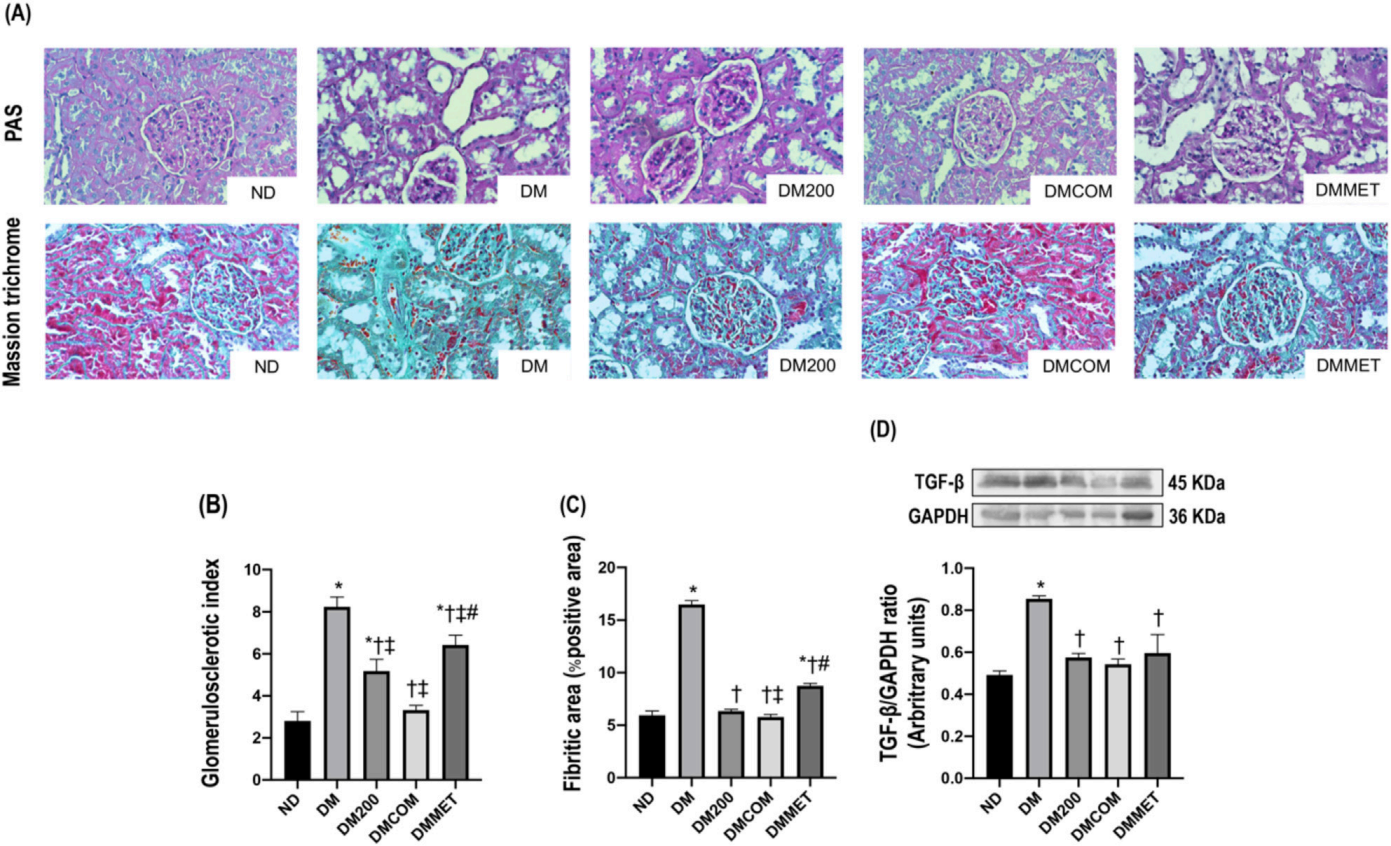
Effect of CN extract on renal fibrosis. Histological sections of kidneys stained with PAS and Masson’s trichrome (x40) **(A)**, Glomerulosclerotic index **(B)**, Fibrotic area **(C)**, Western blot analysis showing the renal expression of TGF-β **(D)**. ND: normal rats treated with vehicle; DM: diabetic rats treated with vehicle; DM200: diabetic rats treated with CN extract 200 mg/kg/day; DMCOM: diabetic rats treated with CN and metformin 100 mg/kg/day; DMMET: diabetic rats treated with metformin 100 mg/kg/day. Values with different superscript letters *p < 0.05 versus ND; ^†^ *p* < 0.05 versus DM; ^‡^ *p* < 0.05 versus DM200; ^#^ *p* < 0.05 versus DMCOM (LSD test, *p* < 0.05).

The reduction in renal inflammation corresponded to improvements in renal morphology. H&E staining (Figure 7) revealed macrophage infiltration, renal tubular dilation, increased Bowman’s capsule area, periglomerular fibrosis, and interstitial fibrosis. The combination treatment significantly reduced renal histopathological lesions and the semi-quantitative kidney injury score in the DMCOM group in comparison to the DM group (Figures 7A, B) (*p* < 0.05). Furthermore, the nephrin expression, which lower in the DM group than in the ND group, appeared to be restored in all treatment group (Figure 7C). Similarly, KIM-1 overexpression in DM group was significantly reduced in treatment groups (Figure 7D). PAS and Masson’s trichrome staining demonstrated substantially elevated glomerulosclerosis levels in kidney sections of DM rats (Figure 8A). The glomerulosclerotic index and fibrotic area were significantly higher in DM groups compared to ND group (*p* < 0.05) (Figures 8B, C). Additionally, DM rats exhibited marked increases in TGF-β, expression compared to the ND rats (*p* < 0.05) (Figure 8D). Notably, the glomerulosclerotic index and fibrotic area were significantly lower in DMCOM group compared to DM group, with a concurrent reduction in TGF-β expression (*p* < 0.05).

## Discussion

Diabetes and its related complications represent as significant public health problem, where single-drug treatment may be insufficient to control the disease progression and reduce the risk of serious complications. Combination therapy is therefore required, as chronic hyperglycemia and its associated complications result from defects in multiple pathways (Sena et al., 2010). This approach involves the use of two or more oral agents or injectables. The coadministration of synthetic antidiabetic drugs with herbs/bioactive compounds is occasionally employed as an adjunct to conventional treatments, particularly enhancing herb-drug antioxidant activity (Tran et al., 2020).

In our experiment, STZ injection combined with a high-fat diet in rats resulted in numerous adverse consequences, including impaired glucose tolerance, dyslipidemia, heightened oxidative stress, ER stress, and renal dysfunction in the DM group (Laorodphun et al., 2024). Additionally, there was a significant increase in body weight and visceral fat weight. Impaired glucose and lipid metabolism contributed to oxidative stress and suppression of the oxidative defense system. This imbalance between the oxidant and antioxidant mechanism further promoted renal damage, accompanied by upregulation of GRP-78 proteins, calpain-2, CHOP, and caspase-12, indicating renal ER-stress (Laorodphun et al., 2022). Notably, our findings demonstrated that the plasma glucose levels in DM rats were effectively reduced by the combination of CN extract and metformin in the DMCOM group.

*Clinacanthus nutans* (CN) extract has been used in traditional medicine across tropical Asia for decades. This plant is listed by Thai Ministry of Public Health as a primary remedy for treating skin inflammations and lesions caused by virus infection (Thongyim et al., 2023a). Phytochemical investigations of *CN* extract have revealed that the plant contains a diverse array of bioactive compounds (Supplementary Table S1). Notably, glyceryl 1,3-distearate, identified as a fatty acid, is the major bioactive compound in CN extract and plays a key role in its anti-inflammatory activity. Furthermore, studies on CN populations in Thailand have reported the presence of gallic acid and quercetin, both of which contribute to antibacterial and antioxidant activities (Chiangchin et al., 2023). Gallic acid has demonstrated antidiabetic properties in animal studies, showing its ability to lower blood glucose levels and increase insulin sensitivity (Behera et al., 2023). Similarly, quercetin has shown potential as a treatment for type 2 diabetes by enhancing insulin secretion and sensitivity (Dhanya, 2022). Among these, six C-glycosyl flavones have been identified in Thai varieties of the plant, including vitexin, isovitexin, schaftoside, isomollupentin 7-O-β-glucopyranoside, orientin, and isoorientin (Alam et al., 2016). These flavones are considered key contributors to the plant’s medicinal properties. Recently, its broad biological activities have been reported including antioxidant, anti-inflammatory, anti-bacterial, anti-viral, and anti-diabetic effects (Hasnat et al., 2024). Imam and colleagues demonstrated the anti-diabetes activity of CN aqueous leaf extract in streptozotocin-induced type II diabetic (T2D) rats (Umar Imam et al., 2019). Administration of CN aqueous extract at dose of 100, 200 mg/kg/day significantly reduced fasting blood glucose levels post-intervention compared to the untreated group (Umar Imam et al., 2019). Additionally, the extract decreased total cholesterol levels and F2-isoprostane, a marker of oxidative stress marker (Umar Imam et al., 2019). In our study, using a different extract solvent, CN ethanolic extract exhibited a potent effect in lowering fasting plasma glucose levels. Moreover, its combination with metformin significantly enhanced the ability to control plasma glucose levels, along with improvements in related lipid metabolism parameters, such as visceral fat weight, plasma cholesterol, plasma triglyceride, plasma HDL, and plasma LDL levels.

Due to the imbalance in lipid metabolism, which may contribute to oxidative stress and ER stress, leading to renal damage and, eventually, renal dysfunction, we investigated the effect of CN extract and its combined effects on reducing oxidative stress, renal ER stress and the associated abnormalities or damage (Chae et al., 2023). Histological analysis of the renal tissues of DM rats revealed significant alterations, including macrophage infiltration, dilatation of Bowman’s capsule, and fibrosis in both the glomerular and tubular regions. These pathological changes were mitigated in DMCOM group, treated with the combination of CN extract and metformin, suggesting the therapy’s potential to protect against renal damage. Additionally, reductions in the upregulation of TGFβ and KIM-1 proteins were observed, indicating improvements in glomerulosclerosis and tubulointerstitial fibrosis-key pathological features of kidney diseases. Mechanistically, the strong antioxidant activity of CN extract may play a crucial role in renal protection. It is well-established that oxidative stress is a significant initiator and primary contributor to ER stress (Laorodphun et al., 2024). The accumulation of ROS in the ER of proximal tubular cells can induce excessive ROS production, triggering ER stress, and the subsequent ER stress response (ESR) and redox imbalance (Cao and Kaufman, 2014). In this study, considerable increases in calpain-2, caspase-12, GRP78, and CHOP levels were observed in the renal cortical tissue of DM rats, indicating pronounced renal ER stress. However, all these markers of ER stress were significantly improved in the DMCOM group. Additionally, we observed an increase in glutathione (GSH) concentration and a simultaneous reduction in MDA levels in renal tissue. These findings suggest that the combined administration of CN extract and metformin enhanced the expression of defense mechanisms and their associated signaling molecules, including GSH, in response to renal oxidative injury (Laorodphun et al., 2024). This highlights the therapeutic potential of CN extract in mitigating renal ER stress and oxidative damage.

These findings align with a previous report demonstrating that CN extract protected pancreatic β-cells from apoptosis in STZ-induced rats by suppressing ER stress and inflammation (Susanti et al., 2024). Susanti and colleagues showed that CN extract attenuated oxidative stress and inhibited JNK signaling pathway, which negatively impacts β-cells survival (Susanti et al., 2024). In our study, we observed elevations in inflammation, oxidative stress, ER stress. We demonstrated that the involvement of the NF-κB signaling pathway, which contributes, at least in part, to the observed inflammation. Systemic stress activates NF-κB pathways at the molecular mechanism level, leading to increased levels of proinflammatory cytokines, including TNF-α and IL-6 (Akhter et al., 2023). Our results revealed that the renal inflammatory response was significantly elevated in the DM group, as evidenced by NF-κB nuclear translocation, and marked increases in the expression of kidney proinflammatory cytokines and chemokines, such as TNFα, iNOS, IL-6, and COX-2, compared to the ND group. Single treatment with CN extract or metformin reduced the expression of these inflammatory markers. However, the combination treatment exhibited a significantly greater effect in mitigating renal inflammation. Notably, we have previously reported the anti-inflammatory effects of CN extract and its bioactive compounds through inhibition of NF-κB signaling pathway in various disease models including bovine mastitis (Panya et al., 2020; Thongyim et al., 2023b) periodontitis (Thongyim et al., 2024), and dengue virus infection (Jantakee et al., 2024) (Supplementary Table S1). These findings suggest that CN extract is a promising natural substance with potential applications in multiple disease models where inflammation play a key role in pathogenesis.

For T2D, based on the effects of CN extract on β-cells survival and the findings of our study, the results suggest the systemic therapeutic potential of CN extract to improve metabolic imbalance, protect the β-cells survival, and reduce the risk of complications such as renal dysfunction. Furthermore, when combined with metformin, CN extract enhanced anti-diabetes efficacy, demonstrating improved control of hyperglycemia, hyperlipidemia, oxidative stress, ER-stress, and renal injury. Given the established safety profile of CN extract, as evidenced by its long-standing use in traditional medicine, this combination raises the possibility of application in clinical setting. It is particularly promising for patients with a tendency toward resistance to single-drug treatment. Therefore, the therapeutic effect of CN to rescue diabetic nephropathy in an animal model was revealed by the results of this study. However, additional research, particularly a clinical trial, is required to obtain data on the therapeutic efficacy and safety of the product in humans.

## Conclusion

The results of this study suggest that combining CN extract with metformin therapy may provide significant therapeutic benefits for managing T2D. In a T2D rat model, co-treatment with CN extract and metformin demonstrated several positive effects, including reductions in body weight, visceral fat accumulation, hyperglycemia, hyperlipidemia, and renal injury. These improvements were associated with a reduction in oxidative stress, ER stress-induced inflammation, and renal fibrosis.

## Data Availability

The original contributions presented in the study are included in the article/Supplementary Material, further inquiries can be directed to the corresponding author.

## References

[B1] Abu Khadra K. M. Bataineh M. I. Khalil A. Saleh J. (2024). Oxidative stress and type 2 diabetes: the development and the pathogenesis, Jordanian cross-sectional study. Eur. J. Med. Res. 29, 370. 10.1186/s40001-024-01906-4 39014510 PMC11253486

[B2] Akhter N. Wilson A. Arefanian H. Thomas R. Kochumon S. AL-Rashed F. (2023). Endoplasmic reticulum stress promotes the expression of TNF-α in THP-1 cells by mechanisms involving ROS/CHOP/HIF-1α and MAPK/NF-κB pathways. Int. J. Mol. Sci. 24, 15186. 10.3390/ijms242015186 37894865 PMC10606873

[B3] Alam A. Ferdosh S. Ghafoor K. Hakim A. Juraimi A. S. Khatib A. (2016). Clinacanthus nutans: a review of the medicinal uses, pharmacology and phytochemistry. Asian Pac. J. Trop. Med. 9, 402–409. 10.1016/j.apjtm.2016.03.011 27086161

[B4] Baker C. Retzik-Stahr C. Singh V. Plomondon R. Anderson V. Rasouli N. (2021). Should metformin remain the first-line therapy for treatment of type 2 diabetes? Ther. Adv. Endocrinol. Metab. 12, 2042018820980225. 10.1177/2042018820980225 33489086 PMC7809522

[B5] Behera P. K. Devi S. Mittal N. (2023). Therapeutic potential of gallic acid in obesity: considerable shift. Obes. Med. 37, 100473. 10.1016/j.obmed.2022.100473

[B6] Cao S. S. Kaufman R. J. (2014). Endoplasmic reticulum stress and oxidative stress in cell fate decision and human disease. Antioxid. Redox Signal 21, 396–413. 10.1089/ars.2014.5851 24702237 PMC4076992

[B7] Chae S. Y. Kim Y. Park C. W. (2023). Oxidative stress induced by lipotoxicity and renal hypoxia in diabetic kidney disease and possible therapeutic interventions: targeting the lipid metabolism and hypoxia. Antioxidants (Basel) 12, 2083. 10.3390/antiox12122083 38136203 PMC10740440

[B8] Cheng C. Yuan Y. Yuan F. Li X. (2024). Acute kidney injury: exploring endoplasmic reticulum stress-mediated cell death. Front. Pharmacol. 15, 1308733. 10.3389/fphar.2024.1308733 38434710 PMC10905268

[B9] Chiangchin S. Thongyim S. Pandith H. Kaewkod T. Tragoolpua Y. Inta A. (2023). Clinacanthus nutans genetic diversity and its association with anti-apoptotic, antioxidant, and anti-bacterial activities. Sci. Rep. 13, 19566. 10.1038/s41598-023-46105-z 37949910 PMC10638387

[B10] Dhanya R. (2022). Quercetin for managing type 2 diabetes and its complications, an insight into multitarget therapy. Biomed. and Pharmacother. 146, 112560. 10.1016/j.biopha.2021.112560 34953390

[B11] Fan Y. Lee K. Wang N. He J. C. (2017). The role of endoplasmic reticulum stress in diabetic nephropathy. Curr. Diab Rep. 17, 17. 10.1007/s11892-017-0842-y 28271468

[B12] Halimi S. Schweizer A. Minic B. Foley J. Dejager S. (2008). Combination treatment in the management of type 2 diabetes: focus on vildagliptin and metformin as a single tablet. Vasc. Health Risk Manag. 4, 481–492. 10.2147/vhrm.s2503 18827867 PMC2515409

[B13] Hasnat H. Shompa S. A. Islam M. M. Alam S. Richi F. T. Emon N. U. (2024). Flavonoids: a treasure house of prospective pharmacological potentials. Heliyon 10, e27533. 10.1016/j.heliyon.2024.e27533 38496846 PMC10944245

[B14] Hu H. Tian M. Ding C. Yu S. (2018). The C/EBP homologous protein (CHOP) transcription factor functions in endoplasmic reticulum stress-induced apoptosis and microbial infection. Front. Immunol. 9, 3083. 10.3389/fimmu.2018.03083 30662442 PMC6328441

[B15] Jantakee K. Panwong S. Sattayawat P. Sumankan R. Saengmuang S. Choowongkomon K. (2024). Clinacanthus nutans (burm. F.) Lindau extract inhibits dengue virus infection and inflammation in the Huh7 hepatoma cell line. Antibiotics 13, 705. 10.3390/antibiotics13080705 39200005 PMC11350823

[B16] Jha J. C. Banal C. Chow B. S. Cooper M. E. Jandeleit-Dahm K. (2016). Diabetes and kidney disease: role of oxidative stress. Antioxid. Redox Signal 25, 657–684. 10.1089/ars.2016.6664 26906673 PMC5069735

[B17] Jin Q. Liu T. Qiao Y. Liu D. Yang L. Mao H. (2023a). Oxidative stress and inflammation in diabetic nephropathy: role of polyphenols. Front. Immunol. 14, 1185317. 10.3389/fimmu.2023.1185317 37545494 PMC10401049

[B18] Jin X. Qiu T. Li L. Yu R. Chen X. Li C. (2023b). Pathophysiology of obesity and its associated diseases. Acta Pharm. Sin. B 13, 2403–2424. 10.1016/j.apsb.2023.01.012 37425065 PMC10326265

[B19] Laorodphun P. Arjinajarn P. Thongnak L. Promsan S. Swe M. T. Thitisut P. (2021). Anthocyanin-rich fraction from black rice, Oryza sativa L. var. indica “Luem Pua,” bran extract attenuates kidney injury induced by high-fat diet involving oxidative stress and apoptosis in obese rats. Phytotherapy Res. 35, 5189–5202. 10.1002/ptr.7188 34327741

[B20] Laorodphun P. Chaisen S. Amattat S. Maphet P. Printrakul N. Pandith H. (2024). Sphagnum cuspidatulum extract prevents acute kidney injury induced by high-fat diet and streptozotocin via alleviation of oxidative stress and apoptosis in pre-diabetic rats. Front. Pharmacol. 15, 1464463. 10.3389/fphar.2024.1464463 39502526 PMC11534586

[B21] Laorodphun P. Cherngwelling R. Panya A. Arjinajarn P. (2022). Curcumin protects rats against gentamicin-induced nephrotoxicity by amelioration of oxidative stress, endoplasmic reticulum stress and apoptosis. Pharm. Biol. 60, 491–500. 10.1080/13880209.2022.2037663 35188833 PMC8865128

[B22] Li J. Ni M. Lee B. Barron E. Hinton D. R. Lee A. S. (2008). The unfolded protein response regulator GRP78/BiP is required for endoplasmic reticulum integrity and stress-induced autophagy in mammalian cells. Cell Death Differ. 15, 1460–1471. 10.1038/cdd.2008.81 18551133 PMC2758056

[B23] Manna P. Jain S. K. (2015). Obesity, oxidative stress, adipose tissue dysfunction, and the associated health risks: causes and therapeutic strategies. Metab. Syndr. Relat. Disord. 13, 423–444. 10.1089/met.2015.0095 26569333 PMC4808277

[B24] Morishima N. Nakanishi K. Takenouchi H. Shibata T. Yasuhiko Y. (2002). An endoplasmic reticulum stress-specific caspase cascade in apoptosis: CYTOCHROME c-INDEPENDENT activation of caspase-9 BY CASPASE-12. J. Biol. Chem. 277, 34287–34294. 10.1074/jbc.M204973200 12097332

[B25] Panya A. Pundith H. Thongyim S. Kaewkod T. Chitov T. Bovonsombut S. (2020). Antibiotic-antiapoptotic dual function of Clinacanthus nutans (burm. F.) Lindau leaf extracts against bovine mastitis. Antibiotics 9, 429. 10.3390/antibiotics9070429 32708141 PMC7400556

[B26] Sai-Ut S. Kingwascharapong P. Mazumder M. A. R. Rawdkuen S. (2023). Optimization of polyphenolic compounds from Gossampinus malabarica flowers by microwave-assisted extraction technology. Future Foods 8, 100271. 10.1016/j.fufo.2023.100271

[B27] Sekulic M. Pichler Sekulic S. (2013). A compendium of urinary biomarkers indicative of glomerular podocytopathy. Pathol. Res. Int. 2013, 782395. 10.1155/2013/782395 PMC3845336 24327929

[B28] Sena C. M. Bento C. F. Pereira P. Seiça R. (2010). Diabetes mellitus: new challenges and innovative therapies. Epma J. 1, 138–163. 10.1007/s13167-010-0010-9 23199048 PMC3405309

[B29] Susanti N. Mustika A. Khotib J. (2024). Clinacanthus nutans leaf extract reduces pancreatic β-cell apoptosis by inhibiting JNK activation and modulating oxidative stress and inflammation in streptozotocin-induced diabetic rats. Open Vet. J. 14, 730–737. 10.5455/OVJ.2024.v14.i2.13 38549571 PMC10970118

[B30] Thongyim S. Chiangchin S. Pandith H. Tragoolpua Y. Jangsutthivorawat S. Panya A. (2023a). Anti-inflammatory activity of glyceryl 1,3-distearate identified from Clinacanthus nutans extract against bovine mastitis pathogens. Antibiot. (Basel) 12, 549. 10.3390/antibiotics12030549 PMC10044565 36978416

[B31] Thongyim S. Chiangchin S. Pandith H. Tragoolpua Y. Jangsutthivorawat S. Panya A. (2023b). Anti-inflammatory activity of glyceryl 1,3-distearate identified from Clinacanthus nutans extract against bovine mastitis pathogens. Antibiotics 12, 549. 10.3390/antibiotics12030549 36978416 PMC10044565

[B32] Thongyim S. Wright T. A. Sattayawat P. Kaewkod T. Baillie G. S. Tragoolpua Y. (2024). Clinacanthus nutans extract lowers periodontal inflammation under high-glucose conditions via inhibiting NF-κB signaling pathway. Front. Pharmacol. 15, 1410419. 10.3389/fphar.2024.1410419 39193343 PMC11347419

[B33] Tran N. Pham B. Le L. (2020). Bioactive compounds in anti-diabetic plants: from herbal medicine to modern drug discovery. Biol. (Basel) 9, 252. 10.3390/biology9090252 PMC7563488 32872226

[B34] Umar Imam M. Ismail M. George A. Chinnappan S. M. Yusof A. (2019). Aqueous leaf extract of Clinacanthus nutans improved metabolic indices and sorbitol-related complications in type II diabetic rats (T2D). Food Sci. Nutr. 7, 1482–1493. 10.1002/fsn3.988 31024722 PMC6475753

[B35] Xie R. J. Hu X. X. Zheng L. Cai S. Chen Y. S. Yang Y. (2020). Calpain-2 activity promotes aberrant endoplasmic reticulum stress-related apoptosis in hepatocytes. World J. Gastroenterol. 26, 1450–1462. 10.3748/wjg.v26.i13.1450 32308346 PMC7152521

