# Supplementary material for: Synergistic amelioration of renal oxidative stress, inflammation, and fibrosis by combination of metformin and Clinacanthus nutans leave extracts in a type 2 diabetic rat model
Source: Front Pharmacol. 2025 Apr 29;16:1558341. doi: 10.3389/fphar.2025.1558341 (PMC12069279; doi:10.3389/fphar.2025.1558341)

Supplementary

Supplementary Table 1. Bioactive compounds identified in *Clinacanthus nutans* and their biological properties

Compounds	Biological properties	References
Glyceryl 1,3-distearate	Anti-inflammatory activity	Thongyim et al., 2023
Gallic acid	Antibacterial activity, antioxidant activity, antidiabetic properties, anti-obesity, anticancer	Chiangchin et al., 2023; Uddin et al., 2022; Dludla et al., 2018; Hadidi et al., 2024
Quercetin	Antibacterial activity, antioxidant activity, antidiabetic properties	Chiangchin et al., 2023; Dhanya, 2022
Vitexin	Antioxidant, antithyroid, antitumor, antimicrobial, antibacterial, radioprotective, hypotensive, anti-arteriosclerotic, antispasmodic, antihypertensive, antiglycation, antiviral, antihepatotoxic and anti-inflammatory	Borghi et al., 2013; Lee et al., 2012; de Freitas Marinho et al., 2023
Isovitexin	Antioxidant properties, α-glucosidase-inhibitory activity, anti-melanogenesis	Khole et al., 2014; Shibano et

		al., 2008; Hou et al., 2019
Schaftoside	Anti-inflammatory, antioxidant, and anti-viral activity	Yu et al., 2024; Wang et al., 2020; Yi et al., 2022
Isomollupentin 7-O-β-glucopyranoside	Anti-inflammatory	Chelyn et al., 2014
Orientin	Antioxidant, anti-inflammatory, anticancer, neuroprotective, cardioprotective, antibacteria	Jing et al., 2020; Zhou et al., 2014; Raza Ishaq et al., 2024
Isoorientin	Antioxidant, Anti-inflammatory	Ziqubu et al., 2020

Supplementary Figure 1. Cytotoxicity of *C. nutans* extracts in HEK293T cells.

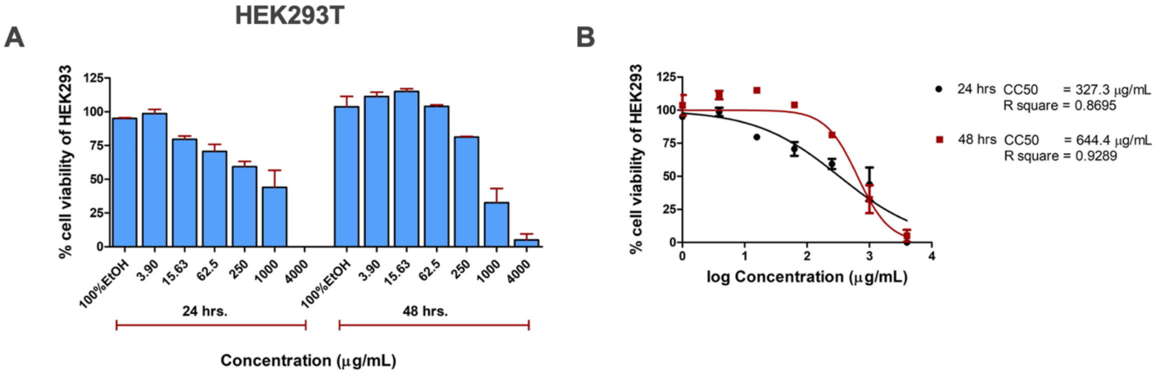

Supplementary Figure 2.

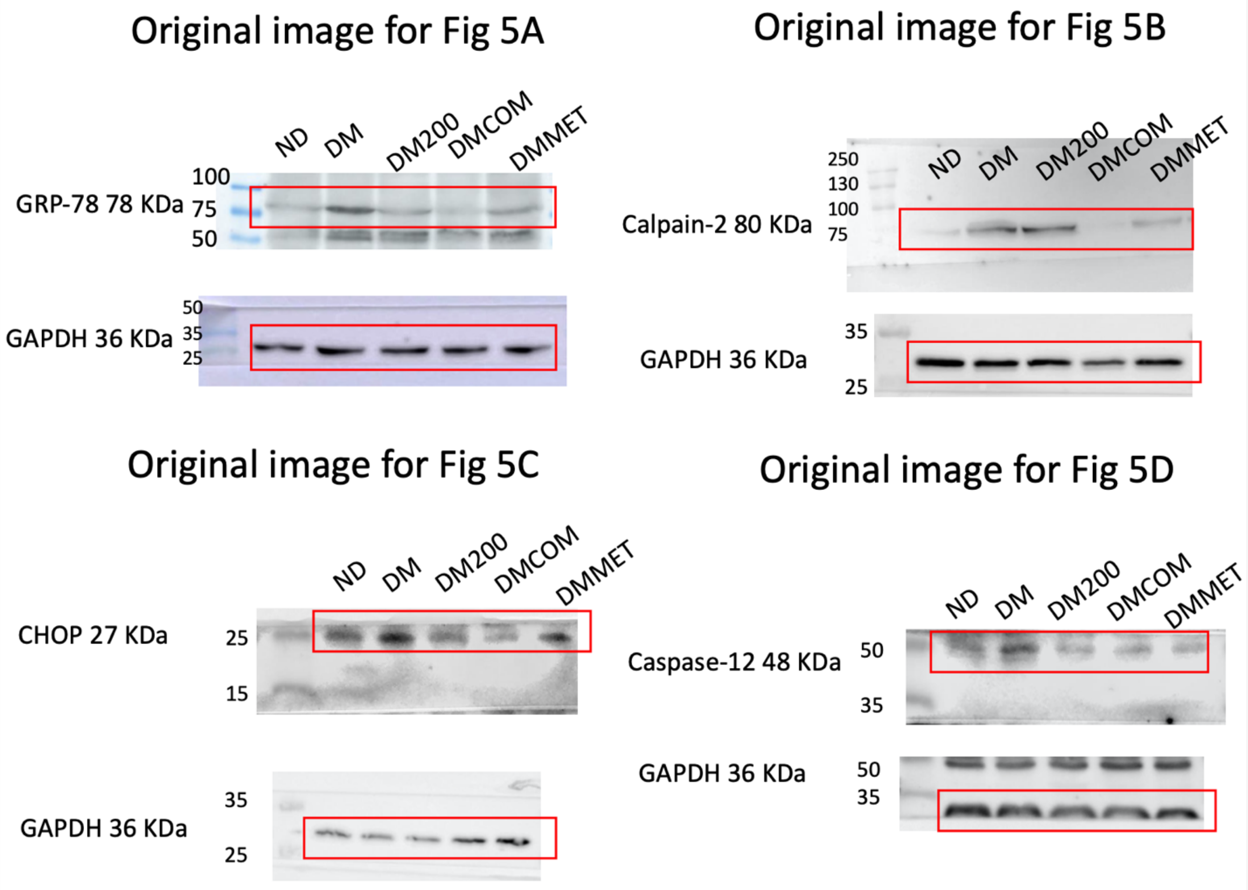

Supplementary Figure 3.

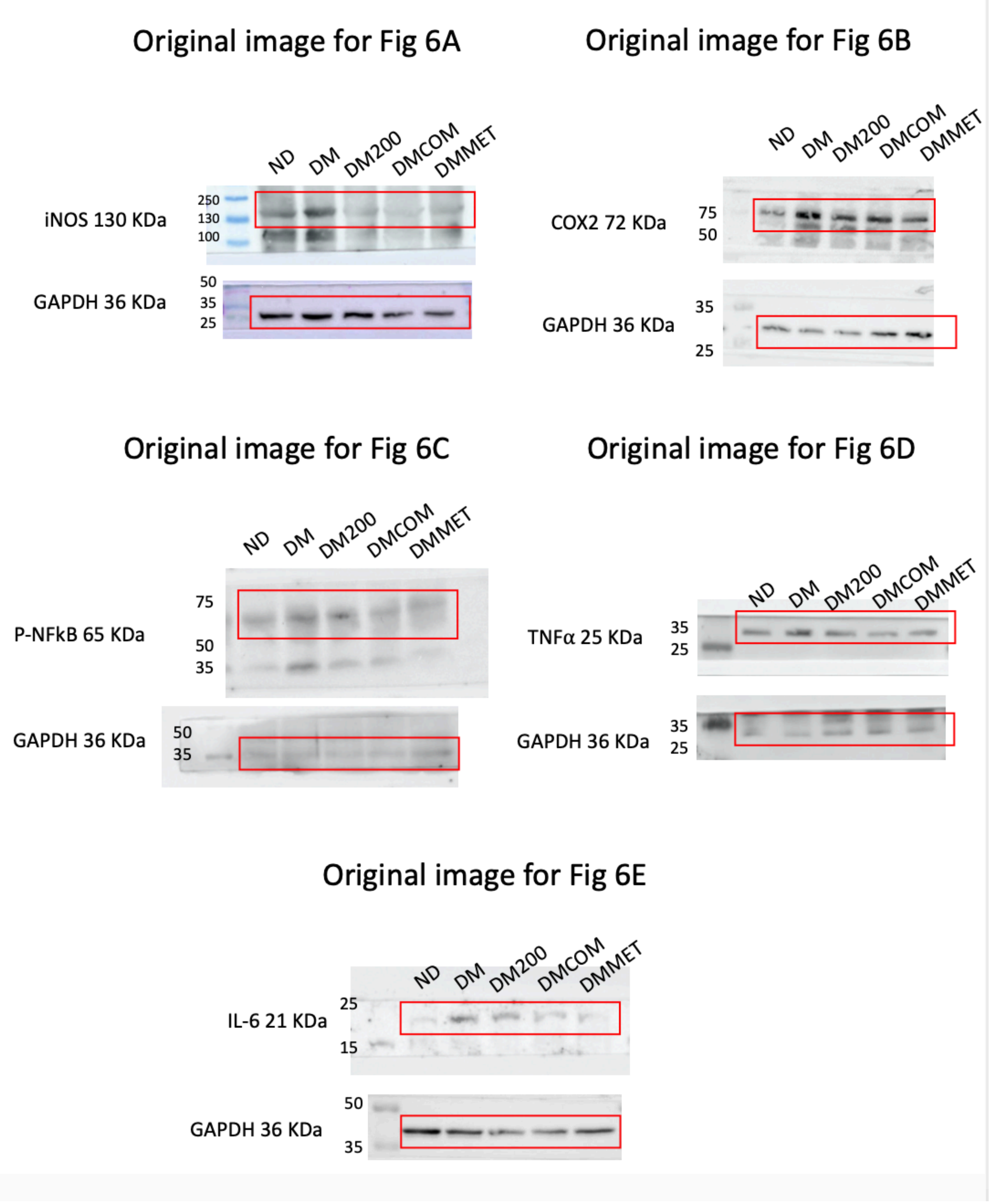

Supplementary Figure 4.

Original image for Fig 7C

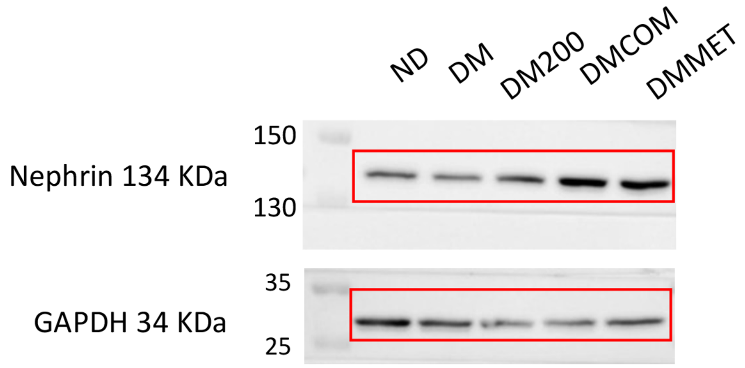

Original image for Fig 8E

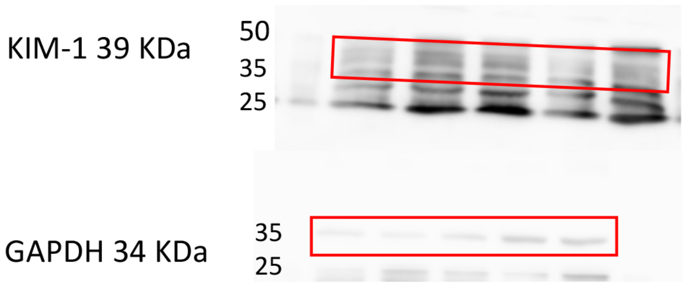

Supplementary Figure 5.

Original image for Fig 8D

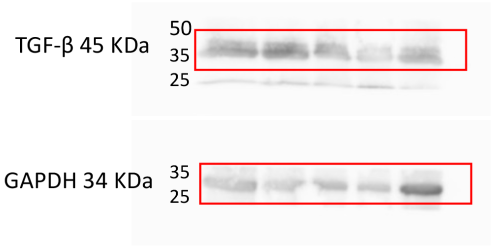

Supplement: Supplementary file 1 [file DataSheet1.pdf]
